# Flavivirus infections and diagnostic challenges for dengue, West Nile and Zika Viruses

**DOI:** 10.1038/s44298-025-00114-z

**Published:** 2025-04-25

**Authors:** Ferralita S. Madere, Aurea Virginia Andrade da Silva, Efemena Okeze, Emma Tilley, Andriyan Grinev, Krishnamurthy Konduru, Mayra García, Maria Rios

**Affiliations:** 1 Office of Blood Research and Review, Center for Biologics Evaluation and Research, US Food and Drug Administration, Silver Spring, MD 20993 USA; 2 Office of In Vitro Diagnostics, Office of Product Evaluation and Quality, Center for Devices and Radiological Health, U.S. Food and Drug Administration, Silver Spring, MD 20993 USA

**Keywords:** Immunology, Virology

## Abstract

Flaviviruses are arthropod-borne viruses, belonging to the *Flaviviridae* family. Dengue virus (DENV), West Nile virus (WNV), and Zika virus (ZIKV) are members of the *Flavivirus* genus, which are primarily transmitted by mosquitos. These viruses result in infections that are predominantly (80–85%) asymptomatic. Clinical manifestations of DENV, WNV, and ZIKV range from mild, self-limiting illnesses, to severe life-threatening diseases for which no therapeutics are available. They have caused extensive outbreaks worldwide, resulting in approximately 400 million cases annually and posing a significant global health threat. Standard diagnostic methods for these viruses rely on nucleic acid test (NAT) and serological assays. However, these approaches have notable limitations. NAT is limited due to the short duration of viremia and low viral loads, which restricts the detection of viral RNA. Serological assays are hindered by antibody cross-reactivity among DENV, WNV, and ZIKV, leading to inaccurate differential results and misdiagnosis. This review provides an overview of DENV, WNV, and ZIKV while addressing ongoing challenges in the differential diagnosis of these viruses, highlighting advancements in technologies. In this review, we aim to increase awareness of the limitations of DENV, WNV, and ZIKV diagnostics. It is our hope that the culmination of these insights will help to facilitate the identification of areas in need of innovation and increased study, which can aid in the development of new approaches to mitigate the global impact of these viruses and improve health outcomes.

## Introduction

Arthropod-borne viruses, commonly known as arboviruses, are viruses that infect and replicate in arthropods and vertebrates. Infected arthropods act as vectors to transmit the virus to vertebrates, which serve as reservoir hosts. These vertebrates subsequently infect other arthropods during blood meals, completing the lifecycle sustained in nature^1,2^. According to the Centers for Disease Control and Prevention (CDC), there are over 550 arboviruses (https://wwwn.cdc.gov/ArboCAT/VirusBrowser.aspx), with approximately 130 known to be human pathogens (https://ndc.services.cdc.gov/case-definitions/arboviral-diseases-neuroinvasive-and-non-neuroinvasive-2015/).

Unlike host-restrictive viruses such as measles or polio, arboviruses can infect a broad range of organisms, including both invertebrates and vertebrates. For instance, WNV can infect different species of mosquitoes *(Culex* and *Aedes*)^3^, ticks (*Ixodidae* and *Argasidae*), and over 150 vertebrates, including mammals that have been found susceptible to WNV infection^4^. Environmental factors are known to play a major role in the transmission and spread of arboviruses by influencing vector distribution and activity. Arboviruses are RNA viruses genetically subdivided into three groups, six families, and ten genera (Table 1). Among these, the *Flavivirus* genus within the *Flaviviridae* family contains over 50 described viruses^5^, eight of which are significant human pathogens. These include six mosquito-transmitted viruses such as DENV, Japanese encephalitis virus (JEV), Saint Louis encephalitis virus (SLE), WNV, yellow fever virus (YFV), and ZIKV, as well as two tick-transmitted viruses, including tick-borne encephalitis virus (TBEV) and Powassan encephalitis virus^5^.

**Table 1:** Arbovirus genome and classification

RNA genome	Family	Genus	Examples
Single-stranded positive-sense	*Togaviridae*	*Alphavirus*	Chikungunya virus
	*Flaviviridae*	*Flavivirus*	Dengue virus, West Nile virus, Zika virus
Single-stranded negative-sense	*Bunyaviridae*	*Orthobunyavirus*	La Crosse virus
		*Nairovirus*	Crimean-Congo hemorrhagic fever virus
		*Phlebovirus*	Sandfly fever virus
		*Tospovirus*	Tomato spotted wilt virus
	*Rabdoviridae*	*Vesiculovirus*	Vesicular stomatitis Indiana virus
	*Orthomyxoviridae*	*Thogotovirus*	Dhori virus
Double-stranded	*Reoviridae*	*Orbivirus*	African horse sickness virus
		*Coltivirus*	Colorado tick fever virus

Flaviviruses have caused global-scale epidemics, leading to significant public health impacts. Despite their impact, effective vaccines are available only for JEV, YFV, and TBEV. The DENV vaccine provides limited protection to children and teens aged 9–16 years (https://www.cdc.gov/dengue/vaccine/index.html) and does not cover all serotypes^6^. DENV, WNV, and ZIKV, have recently caused noteworthy global outbreaks. These virus infections often present with mild, non-specific fever symptoms, making early clinical diagnosis challenging. In some cases, symptoms can progress to severe and even life-threatening conditions, for which specific therapeutics are unavailable. Early recognition of symptoms combined with accurate diagnostic testing can guide timely medical interventions to prevent fatalities. Recent outbreaks of these viruses underscore the urgent need for additional diagnostic methods.

This review provides a focused analysis of the diagnostic challenges associated with DENV, WNV, and ZIKV infections, emphasizing the unique difficulties in clinical and laboratory diagnosis. It highlights recent efforts to advance differential diagnosis while identifying critical gaps that hinder accurate and timely diagnostic process. Thus, it emphasizes the pressing need for innovative solutions to enhance early and accurate diagnosis to assist medical decision for proper patient care.

## Biology

**Fig. 1:**
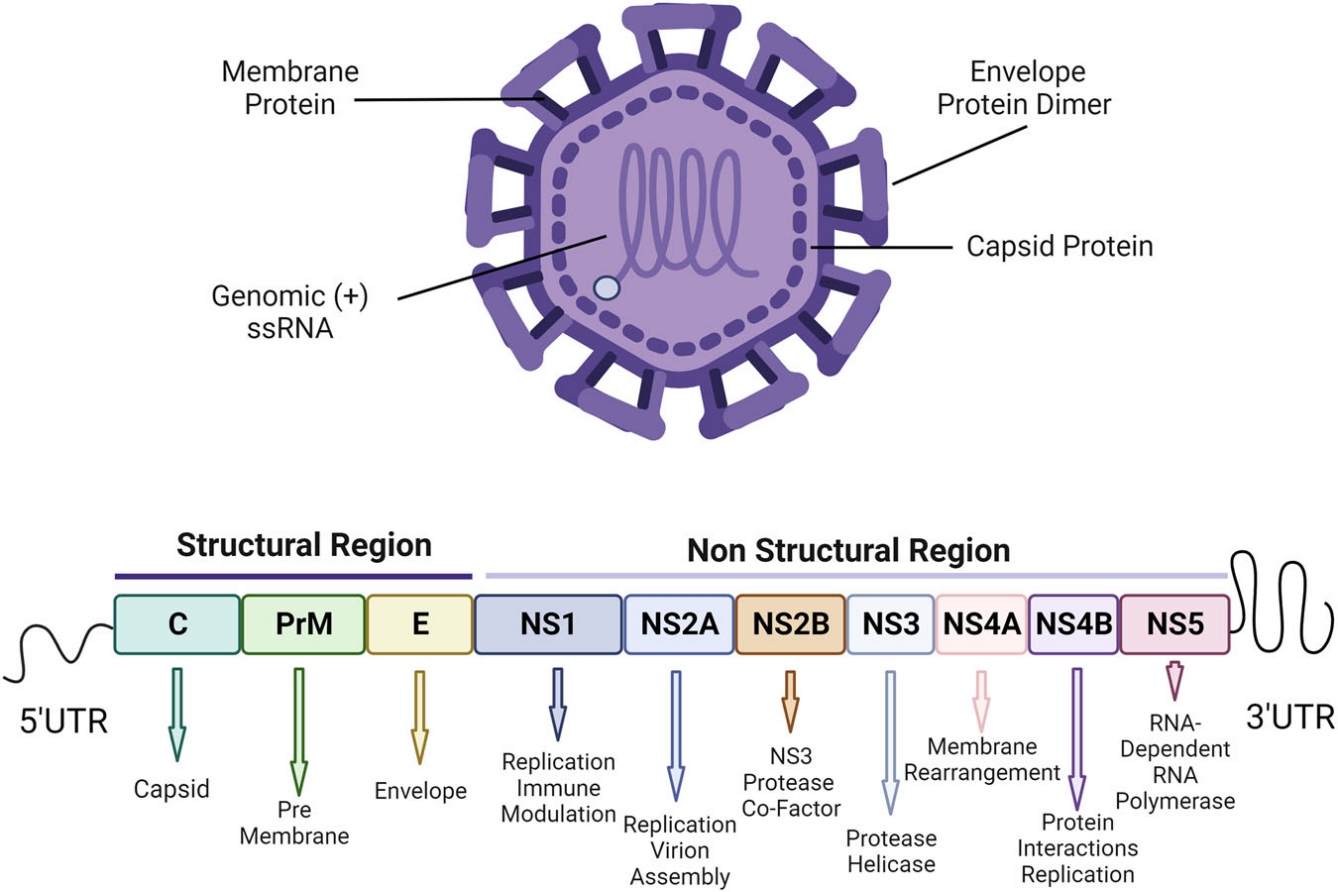
The Flavivirus genome. Flaviviral genomes are about 11 kb in length and encode a single polyprotein that is processed post-translationally by cellular and viral proteases into ten mature viral proteins. The structural regions include capsid (C), pre-membrane (prM) and envelope (E) proteins while the non-structural region includes NS1, NS2A, NS2B, NS3, NS4A, NS4B, NS5 proteins, all which play many diverse roles in the viral lifecycle. Created with Biorender.com.

Flaviviruses are a genus of enveloped positive-sense single stranded RNA viruses belonging to the *Flaviviridae* family^7^ (Table 1). Their genomes are approximately 11 kb and are flanked by a 5’ untranslated region (UTR) with type-I capping and a 3’ UTR that lacks a polyadenylated tail, both forming stem loop structures involved in host immune evasion and viral replication (Fig. 1)^7,8^. A single polyprotein is encoded by the flaviviral genome which is post-translationally processed by cellular and viral proteases into a total of ten mature viral proteins including three structural (capsid (C), pre-membrane (prM), and envelope (E), and seven non-structural (NS) proteins (NS1, NS2A, NS2B, NS3, NS4A, NS4B, NS5)^7,9^. While structural proteins are critical for virion production, attachment and entry, non-structural proteins play many diverse roles in the viral lifecycle, including host immune modulation, viral replication, virion assembly, viral protein translation and processing of the viral polyprotein^8,10^. Viral replication is highly dependent on host factors and requires receptor binding to the E protein, viral entry via endocytosis, release of the viral genome through endosome fusion, viral replication in the endoplasmic reticulum (ER), virion assembly and packaging by way of the trans-Golgi network, and exocytosis to release the mature virion. Flavivirus receptor binding of virions via the E protein can be mediated through several different host plasma membrane proteins including, but not limited to, heparan sulfate, laminin, integrins, heat shock proteins, TIM and TAM proteins and lectins. Preferred viral receptors vary between flaviviruses and are likely determined by E binding protein sequences^11^. Viral entry takes place through clathrin-mediated endocytosis in which endosome pH is lowered causing exposure of the E protein fusion loop and its fusion with the endosome membrane, triggering release of viral RNA into the cytoplasm^12,13^. Supported through the process of ER remodeling, the viral polyprotein is then translated by host ribosomes and cleaved by host and viral proteases to produce viral proteins^7^. The flavivirus assembly process is tightly coordinated with viral replication and is thought to be supported by non-structural proteins NS2A, NS2B and NS3 which play a role in processing of the viral polyprotein, arranging host and viral factors and recruiting the viral genome^14,15^. Viral budding is driven by prM and E glycoproteins and is thought to couple tightly with nucleocapsid formation^14,16,17^. Following assembly, the virion travels from the ER, through the Golgi apparatus where virion maturation occurs through cleavage of the prM-E complex^14^. Virions are transported through the secretory pathway via cytoplasmic vesicles and released through exocytosis where they initiate infection in new host cells^18^.

## Genetic and protein homology

Viruses of the *Flaviviridae* family share high genetic and protein homology^19^. Phylogenetic analysis has revealed that in addition to clustering antigenically, these viruses also cumulate based on the vectors that transmit them^20^. Over ten flaviviral serocomplexes, groups of serotypes with common antigens, have been described with many of them containing prominent human pathogens (e.g., the Japanese encephalitis serocomplex includes WNV; the dengue serocomplex contains DENV serotypes 1–4; and the Spondwenii serocomplex includes ZIKV)^20^. Viruses that belong to the same serocomplex have higher nucleotide sequence and amino acid homology between them compared to those from different serocomplexes^19^. Categorization into the same serotype, such as in the case of DENV, is based on viruses that have amino acid sequence homology that equals to or exceeds 60%^21^. Flaviviruses from different serocomplexes, although similar, are less antigenically homologous. For example, ZIKV possesses 57% amino acid homology with WNV and approximately 54–58% with DENV 1–4 viruses.

The major flaviviral surface antigen, E protein, shares 71–93% amino acid homology within the JEV serocomplex containing WNV, and 61–76% among the DENV serocomplex. On a protein level, E protein shares about 38–51% homology among flaviviruses of different serocomplexes^22^. The E protein contains eleven conserved epitopes with 70–90% homology that are associated with immunological cross-reactivity in NS1, the non-structural protein associated with viral replication and immune modulation^23^. The homology of NS3 and NS5 proteins are significantly higher than other non-structural proteins^24^. These proteins are the most conserved, sharing 71–94% amino acid homology among flaviviruses^23^. There is 80–92% amino acid similarity between ZIKV and WNV NS3 proteins and 76–94% among DENV serotypes. Similarly, NS5 has 81–94% amino acid similarity between ZIKV and WNV and 71–78% among DENV serotypes^24^. As a result, this high degree of genetic and protein homology impacts the accuracy of differential diagnostics among these viruses.

## Transmission

DENV, WNV and ZIKV are primarily transmitted by arthropod vectors, and due to their global spread, they are a major threat to public health (Table 2)^25^. DENV and ZIKV are predominantly transmitted through the bite of infected female mosquitoes of the *Aedes* sp. *(A. aegypti and A. albopictus*)^26^ while WNV is mainly transmitted by female *Culex* sp. mosquitoes (in the U.S. *C. pipens, C. tarsalis and C. quinquefasciatus*^27^*;* and in Europe *C. pipiens and C. modestus*^28^). DENV and ZIKV transmission follow sylvatic and urban cycles^29,30^, however, WNV is primarily maintained in nature through an enzootic cycle between mosquitoes and birds, where birds serve as the primary reservoir^31^. Human infection with DENV, WNV or ZIKV is initiated when female mosquitoes perform bloodmeals, inoculating mature viral particles into the skin where these virions are phagocytosed by highly permissible keratinocytes and Langerhans cells in which they undergo initial rounds of replication^32^. Following this, these flaviviruses target dendritic cells that transport them to lymphoid organs where the viruses complete multiple rounds of replication and mature virions enter the blood circulation^11^. The level of viral replication and infectious virions released into circulation will define the host’s capacity to close the transmission cycle by transmitting the viruses to uninfected arthropod vectors upon feeding.

**Table 2:** Modes of transmission

Modes of transmission			
	Flavivirus species		
	DENV	WNV	ZIKV
Vectorial	Yes^50^	Yes^130^	Yes^131^
Vertical	Yes^132,133^	Yes^134^	Yes^135^
Breastfeeding	Yes^43^	Yes^45^	Possible^64^
Blood transfusion	Yes^36^	Yes^38^	Yes^37^
Organ transplant	Yes^40^	Yes^42^	Yes^41^
Needlestick injuries	Yes^136^	Not reported	Yes^137^
Perinatal	Yes^133^	Not reported	Yes^138^
Sexual	Yes^139^	Not reported	Yes^140^

A reservoir of infection, also known as an amplifying host, refers to a host organism that can sustain viral replication at levels high enough to infect the transmitting vector. For DENV and ZIKV, humans are considered reservoirs as they can support viral replication at sufficient levels to infect the mosquito vector and maintain the lifecycle^33,34^. In the case of WNV, many species of birds also serve as reservoirs: American crows (*Corvus brachyrhynchos)*, House sparrows *(Passer domesticus*) and American robins (*Turdus migratorius*) are responsible for the maintenance and transmission of WNV in the United States. Conversely, human and horse infections do not produce viral loads capable of infecting mosquitoes and are, therefore, considered “dead-end hosts”^35^.

In addition to vector transmission, DENV, WNV and ZIKV can also be transmitted from human to human through exposure to contaminated blood (mucocutaneous, percutaneous and blood transfusion)^36–38^, organ transplantation (bone marrow and solid organs)^39–42^, through breastfeeding or oronasal routes, and via vertical transmission in the case of DENV and ZIKV (Table 2)^43–45^. Although rare, there is evidence that WNV can cross the placental barrier in pregnant women, causing infection of the fetus and the development of chorioretinitis, meningitis, and encephalitis^46^. ZIKV is the most widely recognized sexually transmitted arbovirus where male-to-female transmission occurs more frequently than female-to-male^47^. This is likely due to ZIKV being reported to persist primarily in semen^48^, compared to other body fluids. Although rare, nucleic acids of DENV and WNV have been detected in reproductive tract secretions indicating the potential for sexual transmission of these viruses^47^.

## Epidemiology

DENV, WNV and ZIKV are globally distributed with varying outbreak patterns between continents^25^. DENV and ZIKV are classified as neglected tropical diseases due to their negative health, social and economic impact (https://www.paho.org/en/topics/neglected-tropical-and-vector-borne-diseases). Accurate and timely reporting of clinical cases is a significant challenge for many public health organizations, which has led to global incidence being severely unreported. Proper incidence reporting of DENV, WNV and ZIKV is critical for establishing health measures and guidance for potential prevention of new outbreaks. The persistence of these infections and mortalities prompts the need for continued public health surveillance and improved diagnostic testing for these viruses.

### DENV

The first reported outbreaks of DENV are thought to have occurred in Jakarta, Indonesia and Cairo, Egypt in 1779, and as it spread it eventually reached North America (Philadelphia, Pennsylvania) in 1780^49^. Over the 19^th^ century, epidemics continued to occur in East and West Africa. Post-World War II, Southeast Asian nations saw epidemics of DENV hemorrhagic fever, markedly impacting Indonesia^49^. Global cases of DENV infection have been exponentially increasing over the past 50 years with more than 100 countries being affected with outbreaks every year^50^. Factors such as increased urbanization, deforestation and climate change have all contributed to this rise in cases^25,51^. In 2019, the number of DENV cases in the Americas reported to the Pan-America Health Organization (PAHO) accounted for 3.1 million cases of disease, including 28,217 severe DENV infections and 1823 deaths. In 2023, the reporting on DENV cases in the Americas increased to 4.6 million, 3.4 times higher than in 2019, but was accompanied by a drop in severe DENV infections to 7954, and an increase in fatalities to 2423. Since 2010, DENV has been a notifiable disease in the U.S., with a total incidence rate of 203 cases of DENV infection per 100,000 people in 2023, highlighting the ever-present public health concern that DENV poses both nationally and globally^52^. Between the years of 2019 and 2024 there was an increase in DENV cases in the Americas of 19.4%. DENV outbreaks in 2024 have reached unprecedented levels, affecting 90 countries with cases exceeding 7.6 million and leading to a death toll of 3000 people by April, when WHO declared it a global threat to public health and classified the outbreak as a “Multi-Country Grade 3 Outbreak” (https://www.paho.org/en/topics/dengue/dengue-multi-country-grade-3-outbreak). As of January 2, 2025, PAHO has reported over 12.84 million of cases in the Americas in 2024, where 0.17% represented severe disease cases with a case fatality rate (CFR) of 0.061% (https://www3.paho.org/data/index.php/en/mnu-topics/indicadores-dengue-en/dengue-regional-en/261-dengue-reg-ano-en.html).

### WNV

WNV human infection was first reported in the West Nile district of Uganda during an epidemiological investigation for yellow fever virus in 1937^53^. After WNV identification, a sequence of outbreaks occurred in Israel (1950s) and Europe (1962). It was not until the 1990s when WNV outbreaks occurred across Africa, Asia, Australia, Europe, and the Middle East^25^. The first major European epidemic of WNV occurred in Romania in 1996. Closely following this, WNV emerged in the United States in 1999, causing an outbreak of 62 cases of which 59 (95.16%) were neuroinvasive, and 7 (11.29%) led to fatalities^54^. This outbreak also resulted in a multitude of deaths in avian and equine populations^55^. WNV has caused three major outbreaks in the U.S.: first in 2002 there were 4156 confirmed cases including 2946 (70.88%) cases of neurological disease and 284 (6.83%) deaths^56^; second in 2003 there were 9862 cases, with 2866 (29.06%) neurological manifestations and 264 (2.67%) deaths; and the third outbreak occurred in 2012, with 5674 cases confirmed including 2873 (50.63%) cases of neurological diseases and 286 (5.04%) deaths. From 1999 to 2023, a total of 58,981 confirmed cases of WNV disease were reported to the CDC, including 30,228 (51.25%) cases of severe neurologic disease and 2812 (4.76%) fatalities. The CDC estimates that for every 150 infections, one of them (150:1) will lead to neuroinvasive disease, indicating that there has likely been over 4.5 million WNV infections in the U.S. Conversely, based on statistical data from blood donations and neurological cases reported in North Dakota^57^ it has been estimated that there is one case of neuroinvasive disease for every 256 infections (256:1), which indicates that there has been over 7.7 million WNV infections in the U.S. between 1999 and 2023 based on neuroinvasive cases reported to the CDC ArboNet (https://www.cdc.gov/west-nile-virus/data-maps/historic-data.html). WNV is now endemic in Central and Southern Europe where it causes annual outbreaks^58^. The European Centre for Disease Prevention and Control (eCDC) has reported 7008 confirmed WNV cases of which 5235 (74.70%) cases were neurological disease and 650 (9.27%) lead to fatalities between 2010 and 2023 (https://www.ecdc.europa.eu/en/west-nile-fever/surveillance-and-disease-data). The estimated incidence of WNV-induced neurological disease in Europe during these 14-years is between 0.8 and 1.3 million. However, WNV cases in Europe have increased in the 5-year period between 2019 and 2023 with 2431 cases reported to eCDC, while only 2804 cases were reported in the 9-year period between 2010 and 2018 (https://www.ecdc.europa.eu/en/west-nile-fever/surveillance-and-disease-data).

### ZIKV

ZIKV was first isolated in 1947 from a rhesus macaque in Uganda^59^ with the first reported case of infection in a human occurring in 1952. Since then, ZIKV continued to circulate primarily in Africa causing sporadic outbreaks^60^. The first recorded outbreak of ZIKV outside of Africa occurred in the Yap Islands in 2007, where nearly 73% of the 6892 residents became infected with ZIKV^61^. Between 2013 and 2014 a major ZIKV outbreak was reported in French Polynesia with 100,000 infections^62^, which was followed by its spread to the Americas. In the Americas, the most notable outbreak occurred in Brazil in 2015, where between 440,000 and 1,300,000 cases were estimated to have occurred^63^. During this time, there was a drastic increase in the number of infants born with microcephaly, approximately 4000 cases in the North of Brazil, which was heavily impacted by ZIKV infection. In 2016, the number of ZIKV cases in Brazil reached 273,904, and in 2017 the number was reduced by 79.2% (31,754 cases), likely due to the development of population immunity (https://www3.paho.org/data/index.php/en/mnu-topics/zika-weekly-en/). However, ZIKV continued to spread throughout South and Central America, and by 2016 it was present in 48 countries, accounting for 175,063 confirmed cases. In 2024 ZIKV transmission continues in 59 countries across the globe. Between 2015 and August 2024 PAHO reported over 1 million cases in the Americas. Although decreasing since 2018, there were 213,212 ZIKV cases reported to PAHO between 2019 and August 2024, including over 34,533 cases occurring in 2024, as of August 20, (https://www3.paho.org/data/index.php/en/mnu-topics/zika-weekly-en/), and ZIKV is expected to continue circulating among the human population in the coming years^64^.

## Clinical manifestations of DENV, WNV and ZIKV infections

The majority of individuals infected with DENV, WNV or ZIKV (~80%) remain subclinical (asymptomatic). Clinical cases are characterized by symptom onset following an incubation period after a mosquito-bite, and clinical manifestations are mostly mild, flu-like symptoms^65^. However, mild cases of DENV and WNV have the potential to progress to severe or life-threatening diseases with clinical manifestations unique for each virus. ZIKV presents a significant threat to human reproduction as it causes serious prenatal problems including life-long sequela or fetal death.

### DENV

DENV fever is caused by any of the four DENV serotypes (DENV-1, DENV-2, DENV-3 and DENV-4). Vectoral infection is initiated at the inoculation site where the first round of replication occurs in Langerhans cells (LCs), macrophages and dendritic cells^66^. The virus then disseminates through the blood and lymphatic systems to several organs including lymph nodes, brain, lungs, heart, gastrointestinal tract, spleen, liver and kidney^65^. DENV has an incubation period of 4–10 days post-exposure. Following this, symptoms generally develop in infections with any of the four DENV serotypes and persist for 2–7 days^67^. Immune protection acquired in the first DENV infection is reported to protect against future infections with that same infecting serotype and gives limited protection against other serotypes due to shared (68–78%) sequence similarity, but distinct antigenicity^68^. DENV fever is diagnosed based on the presence of at least two of the following symptoms: nausea, vomiting, rash, aches, and pain in the eyes (typically behind the eyes), muscles, joints, or bones (https://www.cdc.gov/dengue/hcp/clinical-signs/index.html#:~:text=Clinical%20findings%20include%20nausea%2C%20vomiting,%2C%20restlessness%2C%20and%20liver%20enlargement). These symptoms can precede more severe ailments that include abdominal pain or tenderness, persistent vomiting, fluid accumulation, mucosal bleeds, lethargy, liver enlargement and decreases in platelet count^67^. DENV fever can progress to dengue hemorrhagic fever (DHF), a more severe form of DENV fever, within a few hours, leading to shock, internal bleeding, coagulopathy, increased vascular fragility, plasma leakage, thrombocytopenia, DENV shock syndrome (DSS) and death (https://www.cdc.gov/dengue/signs-symptoms/index.html)^69^. The CDC recommends individuals with the following symptoms to immediately seek medical attention: abdominal pain or tenderness, persistent vomiting (at least 3 times in 24 h), mucosal bleeding (nose or gums), vomiting blood, blood in the stool, lethargy, or restlessness (Table 3).

**Table 3:** Clinical manifestations of DENV WNV and ZIKV infection

	Clinical manifestations	DENV^141–143^	WNV^73,144,145^	ZIKV^44,146,147^
Mild clinical manifestations	Fever	Yes	Yes	Yes
	Headache	Yes	Yes	Yes
	Weakness/Fatigue	Yes	Yes	Yes
	Vomiting	Yes	Yes	Yes
	Nausea	Yes	Yes	Yes
	Diarrhea	Not reported	Yes	Yes
	Myalgia (Muscles aches and pain)	Yes	Yes	Yes
	joint pains	Yes	Not reported	Yes
	Arthralgia	Not reported	Yes	Yes
	Rash	Yes	Not reported	Yes
	Maculopapular rash	Not reported	Yes	Yes
	Retro-orbital pain	Yes	Not reported	Yes
	Conjunctivitis (pink eyes)	Yes	Not reported	Yes
Severe clinical manifestations	Encephalitis	Yes	Yes	Yes
	Meningitis	Yes	Yes	Yes
	Myelitis	Yes	Yes	Yes
	Acute flaccid myelitis	Not reported	Yes	Not reported
	Neuropathy	Not reported	Not reported	Yes
	Guillain–Barre syndrome	Yes	Yes	Yes
	Persistent vomiting	Yes	Not reported	Not reported
	Hemorrhagic Fever	Yes	Not reported	Not reported
	Shock Syndrome	Yes	Not reported	Not reported
	Microcephaly or/and congenital formation	Not reported	Not reported	Yes (vertical transmission)

### WNV

WNV infection starts at the inoculation site where the first round of replication occurs in keratinocytes and Langerhans cells (LCs). Infected LCs migrate to draining lymph nodes where they initiate virus dissemination to several organs^70^. WNV symptom onset occurs after an incubation period of 2–14 days but can arise up to 21 days post-inoculation in immunocompromised individuals (https://www.cdc.gov/west-nile-virus/symptoms-diagnosis-treatment/index.html).

WNV fever is a self-limiting, acute febrile illness that typically resolves in days or weeks^71^. Common symptoms include fever, headache, weakness, myalgia, arthralgia, diarrhea and a transient maculopapular rash^71^. Severe disease is estimated to occur in less than 1% of infections^72^. Severe WNV typically manifests in the form of neuroinvasive diseases (WNND), such as encephalitis or meningitis, which are characterized by high fever, tremors, seizures, neck stiffness, muscle weakness, numbness, acute flaccid paralysis, headache, vision loss, disorientation or coma (Table 3)^73^. WNV has also been reported to be associated with poliomyelitis-like syndrome, generalized myeloradiculitis and Guillain-Barré syndrome, an autoimmune condition that can result in prolonged paralysis^73,74^. WNND are more likely to occur in adults over 60 years old or in individuals with medical conditions such as hypertension and diabetes^73^. WNND has high fatality rates and surviving patients suffer long-term health consequences. A survival analysis reported that 40% (157/381) of WNND patients experienced infection-related symptoms for up to 8 years post-diagnosis^75^.

### ZIKV

ZIKV infection is initiated in epidermal keratinocytes, as well as in immature and dermal fibroblasts^76^ followed by dissemination to organs. ZIKV has tissue tropism to the brain, placenta, skin, testis, kidney, and retina^77^. Unlike DENV, ZIKV does not usually cause a sudden onset of clinical symptoms, adding to its difficulty of detection^78^. Mild, self-limiting symptoms of ZIKV can last for days or a week and generally include fever, headaches, maculopapular rash, arthralgia, myalgia, retro-orbital pain, and non-purulent conjunctivitis^79^. Some uncommon symptoms of ZIKV can also include nausea, diarrhea, mucous membrane ulceration, abdominal pain, pruritus, and thrombocytopenia^80^. Rarely, ZIKV has been reported to cause cardiovascular complications^81^ and Guillain-Barré syndrome (Table 3). Of great public health concern is the fact that ZIKV infection in pregnant women, even during asymptomatic infection, can result in trans-placental transmission of the virus to the fetus. This can cause miscarriage, preterm delivery and congenital ZIKV syndrome which includes microcephaly, decreased brain tissue, epilepsy, optic neuropathy (macular scarring, focal pigmentary retinol, congenital contractures, congenital glaucoma), ventriculomegaly, lissencephaly, learning disability, hearing problems and body movement disorder^82^.

## Diagnostic tools and challenges in differential diagnostics

Flavivirus infection can be diagnosed by detecting the virus (viral isolation, nucleic acid, and antigen assays) and/or the pathogen-elicited immune responses (serological methods) (Table 4). Virus detection may be possible at early stages of the disease (acute period); whereas antibody detection is used for diagnosis of viral exposure after a few days of illness. Generally, disease diagnosis can best be achieved by a combination of tests for detection of viral components (RNA, sometimes antigen) and antibody detection (mostly immunoglobulin (Ig) M, although IgG antibody tests may be used in some cases) (Table 5). The purpose of testing (e.g., surveillance, clinical diagnosis) may also influence the assay of choice.

**Table 4:** Overview of laboratory techniques for the detection of human DENV, WNV, ZIKV

Detection	Technique	Advantages	Disadvantages
Virus	Virus isolation	• Confirmatory method	• Transient viremia (up to 5 days p.o.s.)
		• Specific	• Typically, only performed at reference laboratories due to complexity and safety issues
	Nucleic acid detection (RT-PCR, rRT-PCR)	• Specific	• Transient viremia (≤7 days)
		• Sensitive	• Possible false negative and positive results
			• Equipment and trained personnel needed
	Antigen detection (ELISA, RDT)	• Surface or secreted viral protein detection	• Lower sensitivity and specificity than nucleic acid-based tests
		• Easy-of-use	• RDT only available for DENV NS1 Ag
		• Reduced cost	
		• Good resource for laboratories with limited equipment (ELISA, RDT) and the field (RDT)	
		• Rapid results (e.g., less than 30 min) for RDTs	
Human immune response	ELISA/ RDT	• High availability	• Low sensitivity when testing IgM
		• Easy-of-use	• Low specificity, especially with IgG
		• Low cost	• Delayed or retrospective diagnosis when not an RDT
		• Good resource for laboratories with limited equipment (ELISA, RDT) and the field (RDT)	• Complex interpretation due to cross-reactivity (e.g., demonstrate Ig conversion)
		• Rapid results (e.g., less than 30 min) for RDTs	
	Neutralization (VNA; PRNT)	• Used as confirmatory tool	• IgG interference in secondary and tertiary infections
		• Correlates with specific IgGs in blood specimens	• Time consuming, difficult, and expensive^84^
		• Persistent antibodies^85^	• Usually performed in reference laboratories^84^
			• Reproducibility and testing capacity issues

*Ag* Antigen, *DENV* dengue virus, *IgM* Immunoglobulin M, *IgG* Immunoglobulin G, *NS1* Non-Structural protein 1, *p.o.s.* post-onset of symptoms, *PRNT* Plaque Reduction Neutralization Test, *RDT* Rapid Diagnostic Test, *RT-PCR* Reverse Transcription-Polymerase Chain Reaction, *rRT-PCR* Real-time Reverse Transcription-Polymerase Chain Reaction, *VNA* Virus Neutralization Assay.

**Table 5:** Diagnostic testing algorithm

Flavivirus species	Specimen type	0–7 days post symptom onset	>7 days post symptom onset
DENV^a^	Serum	NAT and an IgM antibody test OR NS1 ELISA test and an IgM detection test	IgM ELISA (detectable for 3 month or longer)
WNV^b^	Serum (and CSF if neurological symptoms are present)	IgM (and NAT if immunocompromised patient)	IgM
ZIKV^c^	Serum	NAT in symptomatic non-pregnant patient^d^	IgM with confirmatory PRNT in symptomatic non-pregnant patient^d^

*CSF* cerebrospinal fluid, *DENV* dengue virus, *IgM* Immunoglobulin M, *NAT* nucleic acid test, *NS1* Non-Structural protein 1, *PRNT* Plaque Reduction Neutralization Test, *WNV* West Nile virus, *ZIKV* Zika virus.

^a^https://www.cdc.gov/dengue/hcp/diagnosis-testing/index.html.

^b^https://www.cdc.gov/west-nile-virus/hcp/diagnosis-testing/diagnostic-testing-algorithm.html.

^c^https://www.cdc.gov/zika/hcp/diagnosis-testing/index.html.

^d^For pregnant women and infant testing algorithms and use of urine and CSF please see^c^.

### Infectivity detection

Infectivity detection includes virus isolation and viral inoculation. Virus isolation is the ‘gold standard’ method to confirm the presence of infectious virus, however, it can only be achieved during the acute viremic phase and up to five days following onset of symptoms^30,83,84^. Flavivirus isolation can be performed using mammalian and *Aedes* mosquito cell cultures^83–87^. Inoculation is performed either intrathoracically with adult mosquitoes^85^, or intracranially using suckling mice^83,88^. These techniques are challenging, time-consuming, expensive, and hazardous^86,89^. These assays have been largely replaced by molecular testing and are currently used for confirmatory testing in reference laboratories or as part of investigative surveillance activities and research.

### Nucleic acid detection

Reverse Transcription-Polymerase Chain Reaction (RT-PCR) using virus-specific primers and probes is highly sensitive, specific, and faster than viral isolation^83,84^. NATs are preferred when specimens can be collected within one week of symptoms, since RNA is unlikely to be detected in blood samples (usually represented by serum or plasma) after that period^89^. Due to transient and low levels of viremia, negative RT-PCR results do not exclude infection.

Detection of viral RNA may vary depending on the type of testing sample due to both viral loads and the duration of the virus on the specimen. For instance, in a study where DENV RNA detection was compared among serum, urine, and saliva, researchers found serum to be the best testing sample for up to day 10 post-fever onset; however, after 11 days of fever onset, urine was the best sample to detect DENV RNA^90^. In the case of WNV, RNA has been detected in urine at a higher rate and load than in serum or plasma^91,92^ and for up to a month in urine for neuroinvasive cases^92^. However, it has also been documented that WNV RNA was detected for up to 104 days when whole blood, not serum or plasma, was used as the testing sample for NAT^93^. Extended utility of NAT for detection of ZIKV may be possible due to prolonged viral loads in selected specimens such as urine and semen^25^, or during pregnancy^94^. Some publications report that ZIKV RNA has been detected more frequently or for longer time periods after onset of illness in urine than in serum by RT-PCR^95^. Conversely, others have shown ZIKV RNA to last longer in serum than in urine with a study showing participants having detectable ZIKV RNA in serum after a median time of 14 days and in urine after 8 days^48^. ZIKV was also detected in semen for up to 34 days post-symptom onset but had a low rate of positivity in saliva and vaginal secretions^48^.

Discrimination of viruses by sequencing or isolation is required if pan-flavivirus PCR amplification is used^96^. RT-PCR can also assist in the rapid diagnosis of specific DENV-1, DENV-2, DENV-3 and DENV-4 serotypes during the acute phase^85,97^. Detection of flaviviruses by NAT usually needs well-equipped laboratories and trained personnel^83^, limiting the number of sites able to perform these tests. U.S. Food and Drug Administration (FDA) authorized NATs are available for DENV, WNV, and ZIVK.

### Antigen detection

Antigen tests detect viral proteins in acute samples through antibodies immobilized either on plates (Enzyme-Linked Immunosorbent Assays -ELISAs-) or in strips (Rapid Diagnostic Tests -RDTs-) followed by detection by monoclonal or polyclonal antibodies that result in an enzyme-mediated colorimetric reaction. Secretion of the NS1 protein from infected cells occurs within 24 h of viral infection^98^ allowing for early dengue diagnosis^99^ and its use as a viremic marker^100^. ELISA kits for DENV NS1^30,83,99^ can yield results within a few hours in laboratories with limited equipment. Currently, there is one U.S. FDA-authorized DENV antigen assay, which is an ELISA kit. RDTs, which may be available in some parts of the world, are useful tools for early diagnosis and patient management despite having lower performance compared to ELISAs^83,101^. Dengue RDTs can provide results in 10–15 min^102^ and may be suitable for field settings where authorized. However, they are only available for secreted DENV NS1 and are not serotype-specific^30,83^. Furthermore, RDTs are expensive and not readily available in endemic regions^99^. There are currently no RDTs authorized in the U.S. for diagnosis of DENV.

Current antigen detection methods for DENV rely on detectable levels of secreted NS1, limiting their sensitivity for accurate detection of ZIKV and WNV infections^103^. Publications report low levels of ZIKV NS1 in serum^103,104^ with concentrations of NS1 being predominantly cell-associated and thus, nearly undetectable in serum^104^. The majority of NS1 protein is also retained in the cell in the case of WNV, with release of the antigen being delayed respect to viral particles^105^. In addition, the use of NS1 protein for ZIKV detection poses a risk of cross-reactivity with DENV^106,107^ and the specificity of antigen detection in secondary ZIKV infections is reduced^103^. These factors decrease the usefulness of NS1 antigen detection as a diagnostic test option for ZIKV and WNV in serum.

### Antibody detection

Serological tests to detect flaviviruses are preferentially used due to their ready availability for patient care, epidemiological studies, and evaluation of vaccine and therapeutic candidates^89,108,109^. Antibodies (IgM and IgG) produced soon after infection persist longer in blood than viremia. In a primary immune response, IgM antibodies appear around five days post-infection and can persist from months (ZIKV, DENV)^110,111^ to years (WNV)^112^. Due to transient viremia, negative NAT samples may be tested for IgM to exclude false negative results^113^, since IgM is considered an indicator of recent infection (https://www.cdc.gov/zika/hcp/diagnosis-testing/) whereas IgG appears later in infection, persists longer and may represent past infections^89^. In suspected WNV disease with neurologic symptoms, the presence of IgM in cerebrospinal fluid (CSF) confirms WNV diagnosis (Table 5 and https://www.cdc.gov/west-nile-virus/hcp/diagnosis-testing/diagnostic-testing-algorithm.html).

Accurate diagnosis by IgM detection can be hindered due to low sensitivity of the assays, absence of IgM in secondary infections, as well as persistence of cross-reactive IgGs from previous infections and/or vaccinations^20,89^, further complicating diagnosis^109^. Geographic areas where flaviviruses co-circulate may cause misdiagnosis of infection due to the high degree of sequence and structural homology between the surface proteins^20,86,114,115^ or due to the “original antigenic sin” in secondary infections, where the highest antibody titer is directed against the first infecting virus and not the most recent one^109,116^. Neutralization tests (described below) are frequently used as a confirmatory tool in presumptive positive and inconclusive results (https://iris.who.int/bitstream/handle/10665/204671/WHO_ZIKV_LAB_16.1_eng.pdf)^89,113^.

In contrast to ELISAs that use virus-specific antigens to capture human antibodies, the IgM Antibody Capture Enzyme-Linked Immunosorbent Assay (MAC-ELISA) utilizes an antibody against the µ-chain of IgM to capture IgMs in the sample, with specificity later determined by the addition of a flavivirus-specific antigen. MAC-ELISAs are commonly used in the detection of ZIKV-^95^ and DENV-IgM^30^ for diagnosis and surveillance due to their simplicity and lack of need for sophisticated equipment. Nevertheless, detection of current infections may be challenging due to the persistence of IgM antibodies, and as mentioned above, false negative-results due low or transient IgM in secondary/tertiary infections^85^ and false positive results due to cross-reactivity^85,95^ may happen.

IgG-ELISAs, even while offering higher sensitivity than IgM, exhibit broader cross-reactivity within flaviviruses groups^83^. In the case of DENV, IgG-ELISAs are not useful for diagnosis of dengue cases, particularly in endemic areas, because they could be detecting antibodies from previous dengue serotype infections. Therefore, a suspected dengue case may be positive for IgG antibodies even when not infected with dengue virus. IgG tests may play a role in serological screening of populations to establish prevalence of antibodies or to determine previous dengue exposure in individuals in endemic areas. The potential use of dengue IgG testing for pre-vaccination testing was challenging due to the reported high degree of cross-reactivity with other flaviviruses, which made it more difficult to discern previous ZIKV vs. DENV exposure in countries where both flaviviruses have recently been transmitted. In an attempt to improve the specificity of antibody assays, flavivirus antigens have been modified. In one example, a modified recombinant envelope domain III-based ELISA was developed for the detection of ZIKV IgG antibodies^117^. In another example, E-proteins were mutated in the fusion loop to discriminate ZIKV from DENV antibodies^118^. Finally, mutant envelope virus-like particle antigens have been designed to differentiate WNV from SLE virus IgM antibodies^119^. Recent assessments identified tests with high levels of specificity that could be implemented as part of dengue Dengvaxia vaccine rollout, licensed in the U.S. and other countries for use in individuals with laboratory confirmed previous DENV infection (https://www.fda.gov/vaccines-blood-biologics/dengvaxia). Currently, confirmation of a previous DENV infection can be achieved by following testing recommendations listed by the CDC (https://www.cdc.gov/dengue/hcp/vaccine/testing.html). According to the CDC, screening tests have minimum performance requirements of ≥75% sensitivity and ≥98% specificity. Other vaccines are approved outside of the U.S., including Takeda’s tetravalent dengue vaccine candidate (https://www.statnews.com/2023/07/11/takeda-withdraws-application-for-dengue-vaccine-from-fda/).

Existing alternative tests include antibodies labeled with fluorescent dyes used to increase sensitivity, multiplicity of detection and ease-of-use of the assay. Antibodies and antigens bound to beads or microspheres (Microsphere-based Immunoassays, MIA)^95^ spanning distinct fluorescent regions can be used for simultaneous antigen detection and/or IgM and IgG determination, respectively.

While there are several commercial test kits available in different parts of the world for anti-IgM and anti-IgG antibody detection, it has been noted that, with the exception of tests authorized by the U.S. FDA and potentially regulatory agencies in other parts of the world, and a few tests independently evaluated at the CDC or by the United Nations Children’s Emergency Fund in partnership with the United States Agency for International Development, publicly available information regarding their validation is usually insufficient to evaluate the performance of the tests^85,108,120^. Additionally, WNV and DENV assays developed prior to the ZIKV epidemic may not have been reassessed with samples positive for anti-ZIKV antibodies after the outbreak^88^.

Virus neutralization assays (VNA) and the plaque reduction neutralization tests (PRNTs) are used to confirm the results of conventional serological methods^89,121^. In PRNT assays^86,89,114^, the number of virus-formed plaques signifying infected cells will be reduced by the presence of neutralizing antibodies in the patient’s serum. However, testing conditions can affect lab-to-lab reproducibility of the assay^122^. Furthermore, extensive IgG interference is experienced especially in secondary and tertiary infections^85,121,123^ and with flavivirus cross-neutralizing antibodies^95,124^. To discriminate neutralization between flaviviruses, neutralization of one flavivirus infection (e.g., PRNT_90_) should be shown to be higher than for another^95^. Normally a ratio of ZIKV to either DENV or WNV PRNT titers of at least 4 would confirm a ZIKV infection^96^, although more conservative approaches have been recently proposed for ZIKV in secondary infections as a 4-fold higher PRNT was shown incapable of discriminating anti-ZIKV antibodies from cross-reactive ones^113^. Improved specificity was observed in PRNT assays of secondary flavivirus infection cases using IgG-depleted samples collected between 3 and 12 weeks after exposure^125^. Other modifications of PRNT have been developed to reduce the volume of reagents and subjectivity of plaque counting, while increasing the number of tested samples; these modifications include colorimetric measurement of viral antigen in microneutralization assays^126^ as well as viruses modified with Green Fluorescent Protein^127,128^ or Luciferase reporter genes^129^.

Additional methods frequently used in DENV cases are hemagglutination inhibition (HI) and complement fixation (CF). HI, while sensitive and relatively easy to perform, is not flavivirus- or serotype-specific^85,97^ and requires acute/convalescent paired serum^30,83,97^. Measuring consumption of complement is limited by the requirement of highly trained personnel and the level of difficulty associated with running these assays^85,97^.

### Closing considerations

The ongoing threat of flaviviruses highlights the need for a comprehensive understanding of their biology, genetics, transmission, epidemiology, pathogenesis, and for the development of accurate differential diagnostics. Reliable diagnostic tools are crucial for early diagnosis, proper patient care, and surveillance. Rapid and cost-effective multiplex NATs and serological tests could help distinguish between infections caused by DENV, WNV, and ZIKV. However, current NATs are hindered by the short duration of viremia and low viral loads. Serological assays, while cost-effective and easy to use for surveillance, face challenges due to the high degree of antigenic similarities among these viruses, which leads to antibody cross-reactivity and complications from pre-existing immunity. These factors make it difficult to conclusively identify infections without virologic confirmation^20^. Public health guidelines often recommend combining multiple diagnostic methods to improve accuracy when diagnosing DENV, WNV, and ZIKV. Additionally, accurate diagnosis and patient care should consider integrating patient history, clinical observations, diagnostic test results, and epidemiological data of the site and timing of exposure. However, the co-endemicity of antigenically similar flaviviruses^20,109^ and their fluctuating prevalence further complicates result interpretation. Addressing these challenges can improve diagnostic capabilities and support efforts to mitigate the impact of flavivirus outbreaks.

## Data Availability

No datasets were generated or analyzed during the current study.
